# MiRNAs in milk can be used towards early prediction of mammary gland inflammation in cattle

**DOI:** 10.1038/s41598-022-09214-9

**Published:** 2022-03-24

**Authors:** T. Tzelos, W. Ho, V. Iliadi Charmana, S. Lee, F. X. Donadeu

**Affiliations:** The Roslin Institute and R(D)SVS, University of Edinburgh, Easter Bush, Midlothian, UK

**Keywords:** Diagnostic markers, Animal physiology

## Abstract

Considering the importance of early disease detection for reducing the huge financial and animal welfare impact of bovine mastitis globally, improved tools are urgently needed that can accurately detect early mammary inflammation. MiRNAs have demonstrated value as disease biomarkers, however, their potential for accurately detecting early mammary inflammation has not been examined in detail. To address this, we investigated the association between levels of four inflammation-associated miRNAs (bta-miR-26a, bta-miR-142-5p, bta-miR-146a and bta-miR-223) and CMT scores (0 to 3) obtained from a large number of individual quarter milk samples (n = 236) collected from dairy cows at different lactations (1 to 4). Initial analyses (n = 21 samples) confirmed that the levels of each of bta-miR-142-5p, bta-miR-146a and bta-miR-223 in whole milk were significantly correlated with mRNA levels of known inflammatory markers (*HP, TNF, CXCL8* and *IL1B*) in milk cells (Rho ≥ 0.49, *P* < 0.005). Subsequent analyses (n = 215 samples) revealed a significant effect of CMT score on each of the four miRNAs analysed (*P* < 0.0001), characterised by a progressive increase in miRNA levels in milk as CMT score increase from 0 to > 1. Moreover, a significant effect of lactation number (*P* < 0.01) for bta-miR-26a, bta-miR-142-5p and bta-miR-146a was attributed to higher miRNA levels during lactation 1 than later lactations. Finally, by generating ROC curves we showed that bta-miR-223 and bta-miR-142-5p levels could identify early inflammatory changes in individual quarter milk samples (CMT1) with high accuracy (100% sensitivity, > 81% specificity). Our results provide novel proof of the value of miRNAs as early diagnostic biomarkers of bovine mastitis.

## Introduction

Conservative estimates (https://fil-idf.org/wp-content/uploads/2016/12/FAO-Global-Facts-1.pdf) show that there are approximately 300 million dairy cows worldwide, which produce ~ 600 million tonnes of milk every year. The European Union is the largest milk producer followed by USA and India (https://www.statista.com/statistics/268191/cow-milk-production-worldwide-top-producers/), the latter country having the largest dairy cow population in the world (https://www.statista.com/statistics/869885/global-number-milk-cows-by-country/).

Profitable milk production in dairy herds critically depends on good udder health. Mastitis remains a major problem for the dairy industry globally, resulting annually in large losses from reduced milk production (both quantity and quality), costly treatments and early culling of animals, in addition to contributing significantly to antimicrobial resistance in cattle through widespread dry cow antibiotic treatment^1–3^. Infectious mastitis has a multifactorial aetiology with presentation and course of the disease varying with the primary causative agent(s). Subclinical mastitis is highly prevalent in modern dairy herds and often develops into clinical disease which has an estimated incidence of about 40%^4,5^, occurring with highest frequency during the periparturient period when physiological stress associated with the high energy requirements of gestation and lactation are at their greatest. In this context, the availability of robust diagnostic tools that can identify animals at early stages of disease is crucial to prevent the high costs derived from lost productivity and treatment of clinically and/or chronically diseased animals^2^ as well as to reduce antimicrobial use through early targeted interventions.

Different approaches are being used for monitoring mammary gland health and detecting early inflammation^6^. Somatic cell counting (SCC), directly or through indirect colorimetric quantification often with California Mastitis Test (CMT), in bulk or individual milk samples is the most widely used approach. On the other hand, bacterial analyses by culturing or PCR provide specific information on the pathogen involved. Interpretation of SCC results can be affected by subjectivity as well as natural variation in somatic cell numbers in milk due to parity, stage of lactation, stress, non-mammary disease, and inter-quarter variability^7^. To address this, alternative approaches have been explored or are being developed, e.g. quantification of inflammation related proteins in blood or milk, such as Haptoglobin^6^, and composite approaches for automated systems^8^, although they do not always meet the conditions allowing efficient and affordable implementation in modern farming systems. The fact that the incidence of mastitis in UK herds, for example, has not changed over the last decade^2^ highlights the need for novel, accurate and cost-effective methods for early disease detection.

Expression levels of miRNAs in biofluids can provide readily available, stable and relatively specific disease biomarkers. Diagnostic miRNA applications have been developed for humans, and are being widely explored in livestock^9^. Numerous studies have characterised miRNA responses to mastitis in cattle, and several potential biomarkers of early mastitis have been proposed (see below). In most cases, miRNA profiles in milk fractions and/or peripheral blood after experimental infection of mammary glands with pathogens including *Staphylococcus aureus*^10–12^, *Streptococcus agalactiae*^13^ and *Streptococcus uberis*^14^, were reported. Changes in the expression of large numbers of miRNAs were usually reported although there was limited overlap in the miRNAs identified across studies. Relatively fewer studies reported changes in milk miRNAs associated with naturally occurring mastitis. Lai et al. (2017, 2019) reported differences in expression of selected miRNAs in whey milk from CMT- and CMT + quarters, whereas Srikok et al. (2020) found differences in miRNA levels in whole gland milk reportedly indicative of subclinical or clinical mastitis. To our knowledge, the precise association between milk miRNA levels and different grades of subclinical mammary gland inflammation, as indicated by SCC, and how this may be affected by parity or lactation number, have not been established. Moreover, the actual diagnostic accuracy, if any, of milk miRNA levels for detecting subclinical inflammation at the individual quarter level, as well as their ability to predict disease course, have has not been determined. To address this, we selected a subset of four miRNAs that have been consistently reported to be upregulated in milk or milk components in association with bovine mastitis^10,12–17^, namely, bta-miR-26a, bta-miR-142-5p, bta-miR-146a and bta-miR-223, and analysed their expression in a large set of individual quarter milk samples in order to establish in detail their association with CMT scores as well as their ability to detect early inflammatory changes in the mammary gland.

## Results

### Associations between miRNA and inflammatory marker levels in whole milk and skim milk samples

Initially, we sought to determine whether the levels of the above-indicated miRNAs, henceforth referred to as miR-26a, miR-142, miR-146a and miR-223, were correlated with inflammatory marker levels in milk samples. To do this, we used a small subset of milk samples (whole and skim fractions) collected from individual quarters (21 and 13 samples with CMT = 0 and CMT ≥ 1, respectively) to compare their expression levels of miRNAs with those of mRNAs corresponding to *HP*, *TNF*, *CXCL8* and *IL1B* in matched milk cell samples. These were chosen as classical markers representing different aspects of the body immune/inflammatory response and which can be robustly detected in milk of cows with mastitis^14,18,19^ and, in the case of HP, are used as diagnostic biomarkers of mastitis^6^. As expected, the levels of each inflammatory marker were strongly correlated with CMT score in milk samples (Rho = 0.52–0.64, *P* < 0.005 for all markers). Moreover, as shown on Table 1, levels of all miRNAs but miR-26a in whole milk were strongly correlated with mRNA levels (Rho ≥ 0.5, *P* < 0.005), whereas the same correlations using skim milk were relatively fewer and weaker (Table 1). In addition, significant correlations in miRNA levels between whole and skim milk were present only for miR-223 (Rho = 0.4, P = 0.008). Based on these results, in subsequent experiments we used whole milk samples to study the association between miRNA expression levels and CMT scores in milk.

**Table 1:** Spearman correlations between the expression levels of miRNAs in whole and skim milk and the levels of inflammatory markers in matched milk cell samples.

Sample type	Marker	*HP*	*TNF*	*CXCL8*	*IL1B*
Whole milk	miR-26a	0.405 (0.150)	0.310 (0.182)	0.657 (0.080)	0.641 (0.084)
	miR-142	0.003 (0.494)	< 0.0001 (0.617)	< 0.0001 (0.604)	< 0.0001 (0.636)
	miR-146a	0.003 (0.494)	< 0.0001 (0.640)	< 0.0001 (0.615)	< 0.0001 (0.634)
	miR-223	0.002 (0.515)	0.001 (0.544)	0.001 (0.561)	< 0.0001 (0.575)
Skimmed milk	miR-26a	0.267 (-0.199)	0.093 (-0.297)	0.162 (-0.249)	0.208 (-0.225)
	miR-142	0.034 (0.370)	0.015 (0.420)	0.066 (0.324)	0.028 (0.383)
	miR-146a	0.103 (0.289)	0.071 (0.319)	0.175 (0.242)	0.123 (0.274)
	miR-223	0.02 (0.404)	0.05 (0.344)	0.055 (0.337)	0.045 (0.351)

Data are presented as *P*-value (Rho value).

### Association between changes in levels of miRNAs and CMT scores in whole milk samples across different lactations

**Figure 1:**
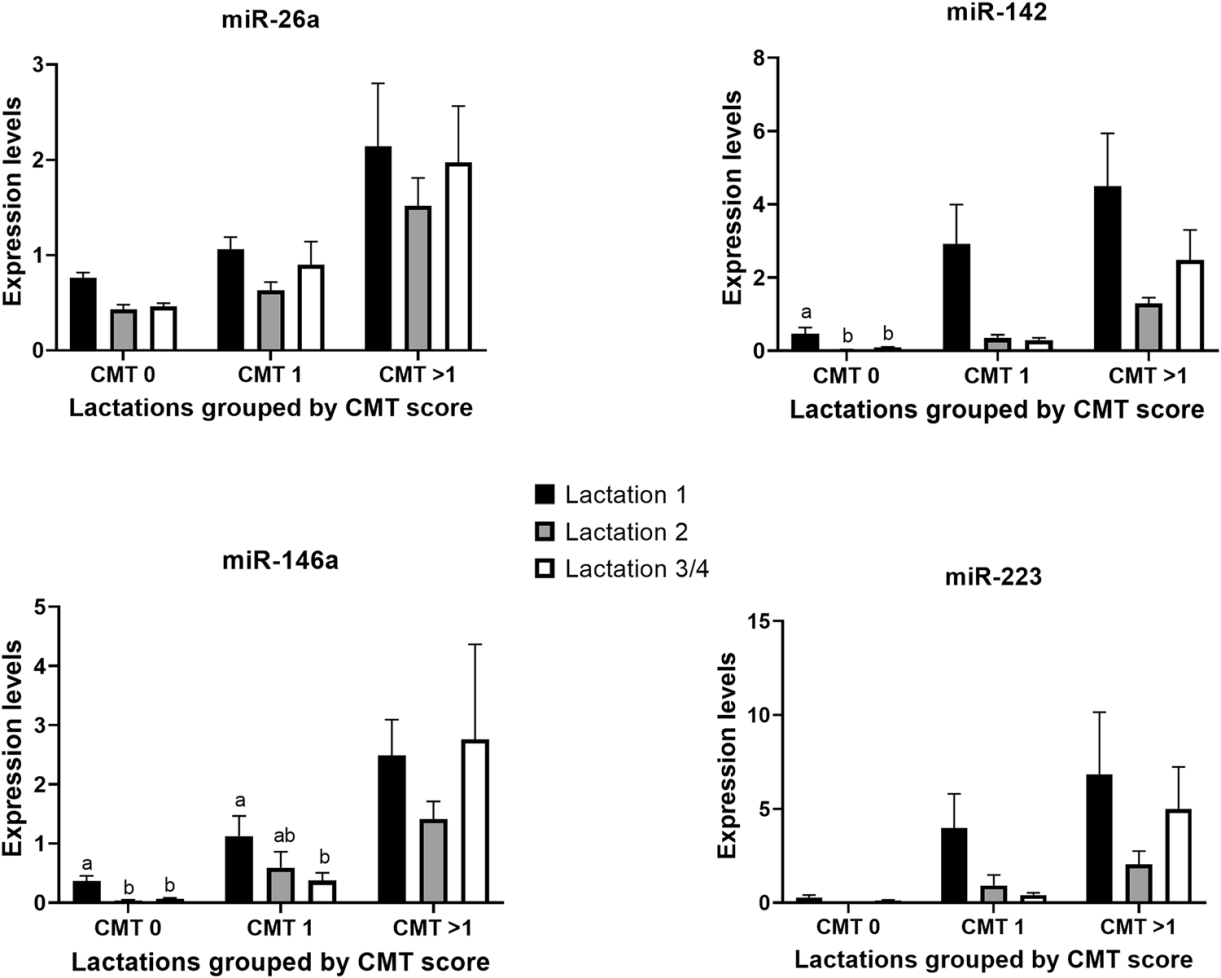
Relative expression levels (Mean ± SE) of bta-miR-26a, bta-miR-142, bta-miR-146a and bta-miR-223 in milk samples with CMT score 0, 1 or > 1 that were collected from individual mammary quarters of cows at different lactations (1, 2, and 3/4). An effect of lactation number was significant for miR-26, miR-142 and miR-146a (*P* < 0.005), for which miRNA levels were higher during lactation 1 than during subsequent lactations (*P* < 0.05). For miR-142 and miR-146a, a CMT x lactation number interaction was also detected (*P* < 0.01); significant differences within CMT scores that were responsible for such interactions are indicated by different letters (a, b).

We examined the response of miRNAs to changes in inflammation in the mammary gland by establishing the association between their levels and CMT scores in whole milk using a large set of individual quarter samples (total, 215) from cows at different lactations (Table 2). Due to the limited number of samples available from cows at lactation 4, data from lactations 3 and 4 were combined for analyses. Results showed that there was a strong effect of CMT score on the levels of all miRNAs (*P* < 0.0001), with mean levels gradually increasing as CMT score increased from 0 to > 1 (Table 3). As shown in Fig. 1, there was also a significant effect of lactation number on each of miR-26, miR-142 and miR-146a (*P* < 0.005), characterised by higher miRNA levels during lactation 1 than subsequent lactations (*P* < 0.05). For miR-223, an effect of lactation approached significance only (P = 0.09). Moreover, a CMT x lactation number interaction was significant for miR-142 and miR-146a, (P ≤ 0.01), due to differences related to lactation number being limited to comparisons within the CMT0 and 1 categories for those two miRNAs (*P* < 0.03). Finally, there were no significant effects (P > 0.05) of either quarter, animal, or day of collection for any miRNAs.

**Table 2:** Numbers of milk samples within each CMT score and lactation number that were used to analyse the associations between miRNA levels and CMT scores in whole milk.

Lactation No	CMT 0	CMT 1	CMT 2	CMT 3	Total
1	35	27	18	2	82
2	37	12	6	4	59
3	32	21	10	1	64
4	6	4	0	0	10
Total	110	64	34	7	215

**Table 3:** Mean (SEM) miRNA levels in milk from mammary gland quarters with different CMT scores (n = 215 samples).

CMT score	bta-miR-26a	bta-miR-142	bta-miR-146	bta-miR-223
0	0.55 (0.030)^a^	0.19 (0.057)^a^	0.15 (0.032)^a^	0.13 (0.049)^a^
1	0.92 (0.111)^b^	1.41 (0.476)^b^	0.73 (0.164)^b^	2.02 (0.797)^b^
> 1	1.94 (0.363)^c^	3.17 (0.762)^c^	2.29 (0.523)^c^	5.18 (1.734)^c^

Means within a column with different superscripts are different (*P* < 0.001).

### Ability of miRNA levels to identify early inflammatory changes in quarter milk samples

**Figure 2:**
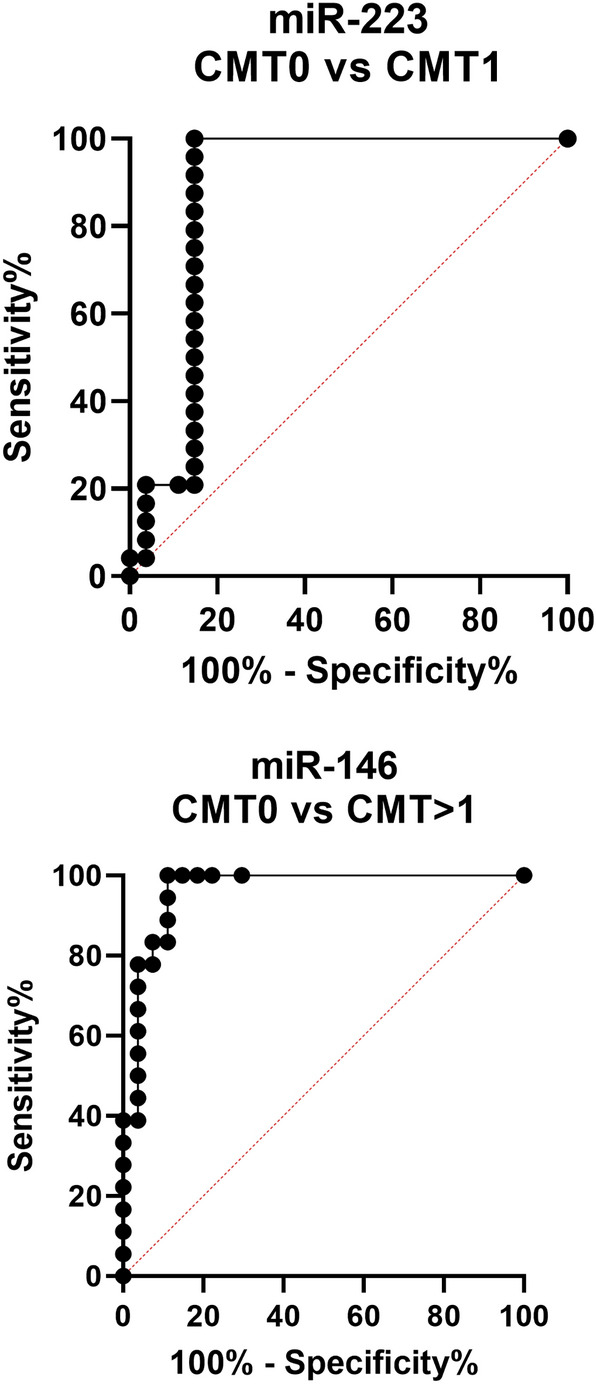
ROC curves obtained from the best performing miRNA predictor for each of the comparisons CMT 0 vs 1 (miR-223) and CMT 0 vs > 1 (miR-146a).

To establish the value of miRNA levels in milk samples as positive predictors of early mammary inflammation, we generated Receiver Operating Characteristic (ROC) curves for all miRNAs using data from samples with CMT 0 *versus* each of CMT 1 and > 1. Best performance data, as indicated by the highest combined value for sensitivity and specificity obtained for each miRNA, are shown in Table 4. The highest sensitivity and specificity for CMT0 *versus* 1 were obtained from miR-223 (100% and 85.19%, respectively; Fig. 2). All miRNAs were more accurate in correctly identifying CMT > 1 than CMT1 samples, with the highest sensitivity/specificity values for CMT0 *versus* > 1 obtained from miR-146a (sensitivity = 100%; specificity = 88.89; Fig. 2). In all cases, miR-26a performed worse than the other three miRNAs.

**Table 4:** Threshold, sensitivity and specificity values for each miRNA based on ROC curve analyses.

Comparison	miRNA	Threshold	Sensitivity%	95% CI	Specificity%	95% CI	Likelihood ratio
CMT 0 *vs*. CMT 1	miR-26a	> 0.1696	91.67	74.15 to 98.52	74.07	55.32 to 86.83	3.536
	miR-142	> 3.787e-005	100	86.20 to 100.0	81.48	63.30 to 91.82	5.4
	miR-146a	> 1.366e-006	100	86.20 to 100.0	70.37	51.52 to 84.15	3.375
	miR-223	> 1.509e-005	100	86.20 to 100.0	85.19	67.52 to 94.08	6.75
CMT 0 *vs.* CMT > 1	miR-26a	> 0.9091	88.89	67.20 to 98.03	92.59	76.63 to 98.68	12
	miR-142	> 0.01432	100	82.41 to 100.0	85.19	67.52 to 94.08	6.75
	miR-146a	> 0.09598	100	82.41 to 100.0	88.89	71.94 to 96.15	9
	miR-223	> 0.01178	100	82.41 to 100.0	85.19	67.52 to 94.08	6.75

Data with the highest combined values of sensitivity and specificity obtained for each miRNA are shown

In addition, we wanted to investigate whether miRNAs could predict a future increase in CMT score in individual quarters. To do this, we used data from animals that were sampled multiple times during the study. Particularly, we compared the miRNA values from non-inflamed quarters (i.e. CMT0) for which two subsequent samples collected 9–11 days apart yielded no change in CMT (Group 1) with those from non-inflamed quarters for which an increase in CMT was observed in at least one of the two subsequent collections (Group 2; see detailed data in Supplementary Table S1). The results showed that, for three out of the four miRNAs, differences in expression levels between quarters in Group 1 and Group 2 were either not significant (miR-26, P = 0.4) or only approached significance (miR-146 and miR-223; *P* < 0.1). In contrast, miR-142 levels were significantly higher (P = 0.03) in Group 2 quarters, although inspection of individual data revealed significant overlap in miRNA values between the two Groups (Supplementary Fig. S1). Finally, when considering clinical outcome, only one cow developed mastitis within the first 4 months after the end of the study. This was an animal for which one of the quarters consistently yielded a CMT score of 3 over four different sampling times during the project.

## Discussion

This is the first study, to our knowledge, to characterise in detail the association between miRNA levels in milk and naturally occurring changes in mammary gland inflammation (as indicated by graded increases in CMT scores of milk samples), as well as the influence of lactation number on those effects. To achieve this, we analysed a much larger number of milk samples than in previous miRNA profiling studies, which we obtained from individual quarters (rather than pooled milk) from cows at different lactations. An important aim of the study was to test the ability of miRNAs in milk to discriminate quarters with different levels of subclinical inflammation and test their ability to predict disease course. To this end, we selected for analyses four miRNAs known to be generically associated with tissue inflammation and to increase in mammary tissues or milk during mastitis. We showed that miRNA levels in milk, most notably miR-223, could be used to discriminate quarters with early inflammatory changes with high accuracy.

Three out of the four miRNAs examined, miR-142, miR-146a and miR-223, are either expressed almost exclusively or highly enriched in several subsets of white blood cells (WBCs) including T- and B- lymphocytes, neutrophils, mast cells and monocytes^20^. In contrast, despite its relative high abundance in WBCs, miR-26a is ubiquitously expressed across the body, being also among the most abundant miRNAs in healthy mammary tissues and milk^21,22^. In line with this, we showed that the levels of miR-142, miR-146a and miR-223 provide more accurate indicators of inflammatory changes in mammary quarter milk than those of miR-26a. Particularly; 1) the levels of all immune mRNA markers analysed in milk cells (*HP, TNF, CXCL8* and *IL1B*) were robustly correlated with milk levels of all miRNAs except miR-26a, and 2) miR-26a displayed lower diagnostic accuracy than the other miRNAs examined, particularly for detecting quarters with mild inflammation (CMT 0 *versus* 1). Since different fractions of milk (skim milk, fat, cells) have been shown to vary in their miRNA content^23^, we decided to analyse both whole milk and a milk fraction devoid of both cells and fat (skim milk). Our finding that the correlation between the levels of miRNAs and immune mRNAs was poor in skim milk compared to whole milk further attests to the predominant immune cell origin of miR-142, miR-146a and miR-223. This is in agreement with the finding that immune miRNAs in general are overwhelmingly associated with the cell fraction of bovine milk^24^, and that changes in their expression, such as detected in the present study, mostly reflect variation in immune cell numbers in milk. In contrast to our findings, a recent study found that levels of miR-146a (and also miR-148a) were reduced in skim milk samples from mastitis compared to healthy cows^17^. The fact that skim milk rather than whole milk was used in that study may explain the discrepancies with our data, and highlight the importance of carefully considering the type of sample when measuring miRNA levels in milk or other biofluids for diagnostic purposes.

We found that, on average, miRNA levels in milk were higher, or tended to be higher, in lactation 1 than in subsequent lactations. Considering that miRNA abundance varies across different leucocyte types, the observed differences may be related to reported differences in the proportions of different leucocyte types in milk between first parity and multiparous cows^25,26^. Alternative, rather than differences in cell numbers, the observed differences in miRNA expression may be related to natural changes in immune status within mammary quarters that in turn may be associated with an increase in the susceptibility of cows to mastitis as lactation number increases^27^. Whichever their cause, the observed effects of lactation number on miRNA levels should be taken into account in the future when investigating the associations between milk miRNAs and mammary gland inflammation and their potential as biomarkers of mastitis, particularly in view that previous studies have often not taken lactation number into account.

Results of ROC curve analyses in our study demonstrate the ability of miRNAs (particularly miR-223, miR-146a and miR-142) to precisely discriminate quarter milk samples with early inflammatory changes. Similar previous analyses showed that miR-146a levels, but also other milk miRNAs not examined in our study such as miR-222 and miR-155, could indeed be used to identify with high accuracy milk samples that scored positive in a CMT test. However, in those studies, distinction between different CMT scores was not made, and as a result, the earliest time at which miRNAs could identify changes in inflammatory status during the course of subclinical disease was not established. In this context, our results provide novel evidence that demonstrates the significant potential of milk miRNAs for early diagnosis of subclinical mastitis, and which could be used to develop novel diagnostic approaches based on single miRNAs or panels of miRNAs. A caveat to this is that we did not find a clear association between miRNA levels and subclinical disease course, based on changes in CMT scores during subsequent collections from the same quarter(s). However, a larger number of samples than used to assess this in our study, as well as data from different dairy farms representing the full spectrum of clinical mastitis prevalence in the sector, should be used in future studies to more accurately determine the potential of miRNAs for predicting subclinical mastitis course in cows. Although SCC is currently used widely for mastitis control, its value for diagnosing subclinical inflammation is limited^7^, and there is an expressed need for improved yet affordable and farmer-friendly approaches for early disease detection by the dairy sector^28^. In this regard, quantification of miRNA levels could have significant value as an early diagnosis tool free of some of the biases associated with SSC results. MiRNA biomarkers might be most useful for control of mastitis when used in combination with other new approaches currently being developed for milk analyses, such as in-line detection tools, allowing on-farm rather than laboratory quantification.

In summary, our novel results show that whole milk levels of the miRNAs, miR-26a, miR-142, miR-146a and miR-223, are positively associated with changes in mammary inflammation as indicated by CMT scores, and that quantification of miRNA levels, particularly miR-142, miR-146a and miR-223, could be used potentially for high-accuracy early diagnosis of subclinical mastitis in cows.

## Methods

### Collection of milk samples

Milk samples were collected during a single lactation from a total of 36 healthy Holstein–Friesian or Holstein–Friesian-cross cows (16 cows were in lactation 1, 9 at lactation 2, 8 at lactation 3, and 3 at lactation 4). From each cow, a milk sample was collected from each quarter on one or several occasions (9–11 days apart), all within 46 to 197 days after calving (Mean ± SE = 112.5 ± 3.78 days). No samples were collected from cows with signs of clinical mastitis (i.e. presence of flakes, clots, or pus in milk, or mammary gland redness, heat or pain) or other obvious signs of disease, or that had recently received intra-mammary antibiotic therapy to treat mastitis. On each occasion, before collection each teat was cleaned with a dry towel, and a small volume of milk squirted out before a milk sample was collected from each quarter into a 50 ml tube. Samples were placed on ice after collection and a CMT test was performed as per manufacturer guidelines (Bovivet CMT liquid, Kruuse; Denmark). Briefly, milk was poured into a CMT paddle and the volume was adjusted to an indicator line in each of the four cups in the paddle. An equal volume of CMT reagent was added to each cup and the samples were mixed for 10 s, after which each sample was given a score of 0 to 3. Fresh milk samples were transported to the laboratory within 1 h of collection. A small volume of whole milk was then directly aliquoted and frozen at − 80 °C. In some instances, the remaining of the sample was centrifuged at 1,900xg for 10 min at 4 °C after which the skim milk fraction was carefully aspirated through the top lipid layer, and further centrifuged at 16,000xg for 10 min at 4 °C to remove residual fat and cell debris before it was frozen at − 80 °C. An additional volume of skim milk was centrifuged at 1,900xg for 10 min at 4 °C to pellet a cell fraction which was then separated and frozen in Trizol at − 80 °C.

All animal procedures were performed with approval from The Roslin Institute (University of Edinburgh) Animal Welfare and Ethical Review Board and following the UK Animals (Scientific Procedures) Act, 1986. The study was carried out in compliance with the ARRIVE guidelines.

All methods were carried our according to required guidelines. A flow diagram of the experimental design can be seen in Supplementary Fig. S2.

### RNA analyses

Aliquots of whole or skim milk (300 μl) were thawed and extracted using Trizol LS (Thermo Fisher Scientific, Waltham, USA), following the manufacturer’s instructions. Before extraction, the samples were spiked with 3.5 μL of exogenous cel-miR-39 (5.6 × 10^8^ copies per sample, Qiagen). The collected RNA was re-suspended in 30 μl of RNase-free water and used immediately or frozen at − 80 °C. A total of 2 μl of RNA was reverse-transcribed in a 10 μL reaction using miRCURY LNA RT Kit (Qiagen) in a Whatman-Biometra Thermocycler (Biometra, USA). The cDNA template was diluted 60-fold and added to 10 μL qPCR reactions which were prepared in 96-well format using miRCURY LNA SYBR Green PCR Kit (Qiagen) and containing primers against bovine sequences for miR-26a (accession no.MIMAT0003516), miR-142-5p (MIMAT0003791), miR-146a (MIMAT0009236), miR-223 (MIMAT0009270) or miR-148-3p (MIMAT0003522), or against cel-miR-39-3p (MIMAT0000010). Template amplification was carried out in an Agilent MX3000P qPCR system (Agilent Technologies, USA). Raw fluorescence data were collected and analysed using MxPro software (Agilent Technologies). Relative transcript abundance was obtained by extrapolating Ct values from a standard curve prepared from a sample pool. Expression levels were separately normalised to the mean expression of bta-miR-148-3p, a highly abundant miRNA in milk^16,24^, and spiked-in cel-miR-39-3p, and the average of the two normalised values was used for final calculations.

Cell samples in Trizol were thawed and extracted following the manufacturer’s protocol. RNA pellets were re-suspended in 10 μL of RNase-free water and used immediately or frozen at − 80 °C until further use. RNA content and quality were determined using the Nanodrop ND-1000 Spectrophotometer (Thermo Fischer Scientific). A total of 500 ng of RNA was reverse-transcribed using SuperScript™ III Reverse Transcriptase (Thermo Fisher Scientific) and mRNA levels were quantified using bovine primers (Table 5) and the SensiFAST™ SYBR Lo-ROX Kit (Bioline Ltd., UK). The cDNA was diluted 1:300 and 2 μl of diluted cDNA and used in 10 μl qPCR reactions. Expression values were separately normalised to CHAMP1 and REPS1 levels and the average normalised value obtained was used for final analyses.

**Table 5:** Primer sequences used for qPCR analyses of inflammatory markers.

Gene symbol	Accession number	Primer sequence (5’-3’)
*CHAMP1*	XM_005213976	**F—**AGCAGTGACCAAGAGCAGGT
		**R—**TCATAGCACGACAGCAACAA
*HP*	NM_001040470	**F—**TCGCTATCAGTGCAAACCCT
		**R—**CCGCACACTGCCTCACATTC
*IL1B*	NM_174093	**F—**AACGTCCTCCGACGAGTTTC
		**R—**CATGCAGAACACCACTTCTCG
*CXCL8*	NM_173925	**F—**AACGAGGTCTGCCTAAACCC
		**R—**TTGCTTCTCAGCTCTCTTCACA
*REPS1*	NM_001354364	**F—**AAGCCGAGAAACATCCAGAG
		**R—**ACATTGGCGGGAGCACTA
*TNF*	XM_005223596	**F—**TGCTGCACTTCGGGGTAATC
		**R-** TTGATGTCGGCTACAACGTG

### Statistical analyses

All statistical analyses were performed using Minitab 18 Statistical Software (Minitab, LLC; USA). Data were assessed for normality using the Kolmogorov-Smirnoff test (P > 0.01) and were log-transformed before analyses if needed. Data were then analysed using one- or two-way ANOVA to test for main effects of CMT score, lactation, and their interaction, with animal, quarter and day of sampling as co-variates, followed by a *post-hoc* Tukey test or, if only two means were compared, Student's t-tests. Spearman’s tests were used to examine correlations involving levels of different RNAs and CMT scores in milk. ROC curve analyses of miRNA data were performed using GraphPad Prism 9 (GraphPad software, San Diego, CA). In all cases, significance was considered at *P* < 0.05.

## Supplementary Information

Supplementary Information 1.

Supplementary Information 2.

Supplementary Information 3.
