# Supplementary material for: MiRNAs in milk can be used towards early prediction of mammary gland inflammation in cattle
Source: Sci Rep. 2022 Mar 24;12:5131. doi: 10.1038/s41598-022-09214-9 (PMC8948199; doi:10.1038/s41598-022-09214-9)
Supplement: Supplementary file 1 — Supplementary Information 1. [file 41598_2022_9214_MOESM1_ESM.docx]

Supplementary Table 1. CMT scores of milk samples (n=19 cows) that were used to test whether miRNA levels could predict a future increase in the CMT score of individual quarters. Scores profiles in samples collected on sequential days (0, 10, 19 and 31) from each quarter are shown. CMT scores in Group 1 quarters remained unchanged throughout the sampling period whereas scores in Group 2 quarters increase after D0. NA-data not available

| **Quarter ID** | **Group** | **D0 CMT score** | **D10 CMT score** | **D19 CMT score** | **D31 CMT score** |
| --- | --- | --- | --- | --- | --- |
| **1** | Group 1 | 0 | 0 | 0 | NA |
| **2** | Group 1 | 0 | 0 | 0 | NA |
| **3** | Group 1 | 0 | 0 | 0 | NA |
| **4** | Group 1 | 0 | 0 | 0 | 0 |
| **5** | Group 1 | 0 | 0 | 0 | 0 |
| **6** | Group 1 | 0 | 0 | 0 | 0 |
| **7** | Group 1 | 0 | 0 | 0 | NA |
| **8** | Group 1 | 0 | 0 | 0 | NA |
| **9** | Group 1 | 0 | 0 | 0 | 0 |
| **10** | Group 1 | 0 | 0 | 0 | 0 |
| **11** | Group 1 | 0 | 0 | 0 | 0 |
| **12** | Group 1 | 0 | 0 | 0 | 0 |
| **13** | Group 1 | 0 | 0 | 0 | NA |
| **14** | Group 1 | 0 | 0 | 0 | NA |
| **15** | Group 1 | 0 | 0 | 0 | NA |
| **16** | Group 1 | 0 | 0 | 0 | 0 |
| **17** | Group 1 | 0 | 0 | 0 | 0 |
| **18** | Group 1 | 0 | 0 | 0 | 0 |
| **19** | Group 1 | 0 | 0 | 0 | 0 |
| **20** | Group 1 | 0 | 0 | 0 | 0 |
| **21** | Group 1 | 0 | 0 | 0 | 0 |
| **22** | Group 1 | 0 | 0 | 0 | NA |
| **23** | Group 2 | 0 | 2 | 2 | 2 |
| **24** | Group 2 | 0 | 2 | 0 | 0 |
| **25** | Group 2 | 0 | 3 | 2 | 2 |
| **26** | Group 2 | 0 | 3 | 3 | NA |
| **27** | Group 2 | 0 | 3 | 2 | 2 |
| **28** | Group 2 | 0 | 1 | 1 | NA |
| **29** | Group 2 | 0 | 1 | 1 | 1 |
| **30** | Group 2 | 0 | 1 | 1 | NA |
| **31** | Group 2 | 0 | 1 | 1 | NA |
| **32** | Group 2 | 0 | 1 | 1 | NA |
| **33** | Group 2 | 0 | 0 | 1 | NA |
| **34** | Group 2 | 0 | 1 | 0 | 0 |
| **35** | Group 2 | 0 | 1 | 0 | 0 |
| **36** | Group 2 | 0 | 1 | 0 | 0 |
| **37** | Group 2 | 0 | 0 | 1 | NA |
| **38** | Group 2 | 0 | 0 | 1 | NA |
