# Supplementary figures and images for: MiRNAs in milk can be used towards early prediction of mammary gland inflammation in cattle
Source: Sci Rep. 2022 Mar 24;12:5131. doi: 10.1038/s41598-022-09214-9 (PMC8948199; doi:10.1038/s41598-022-09214-9)

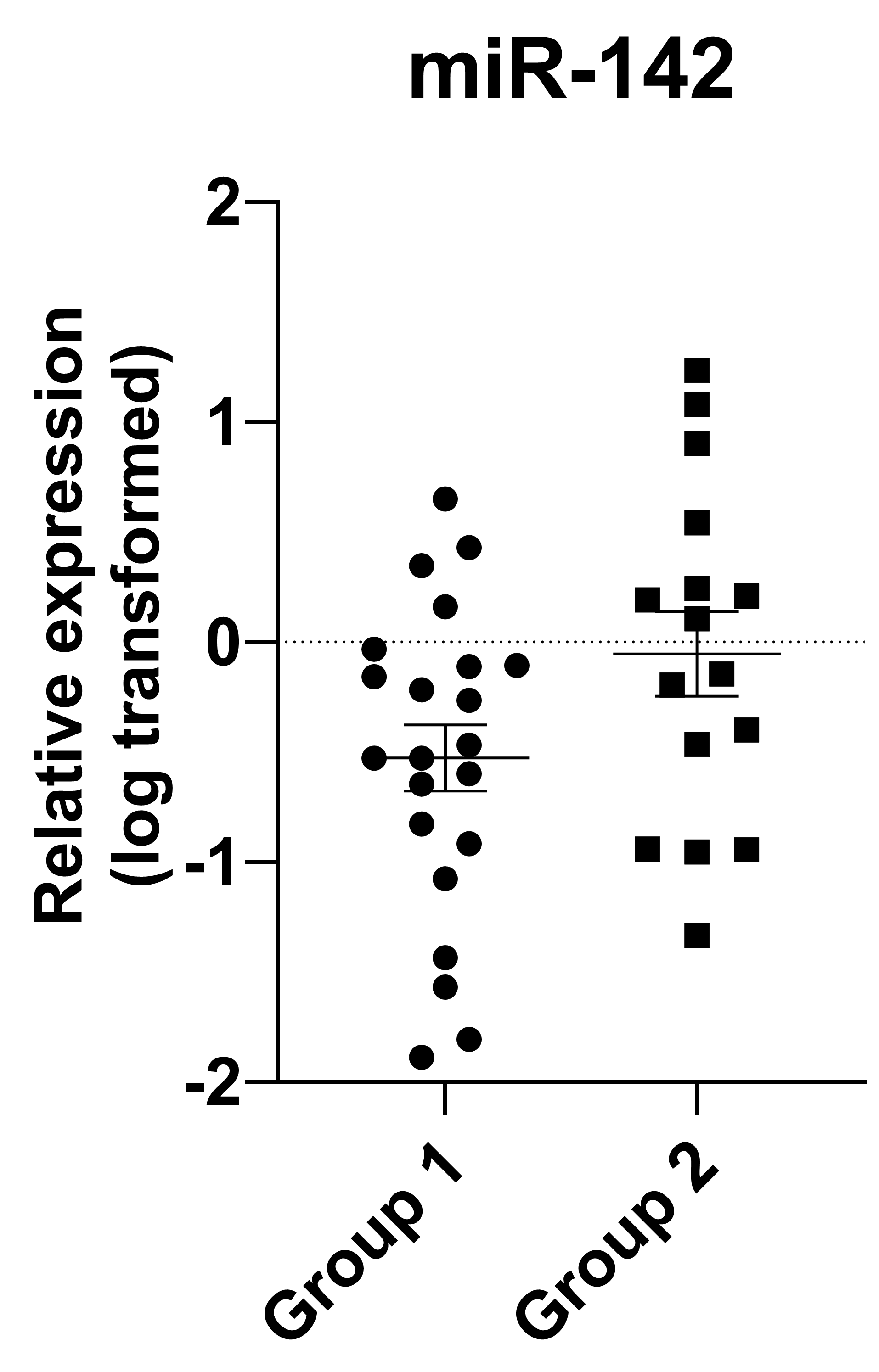

Supplement: Supplementary file 2 — Supplementary Information 2. [file 41598_2022_9214_MOESM2_ESM.tif]

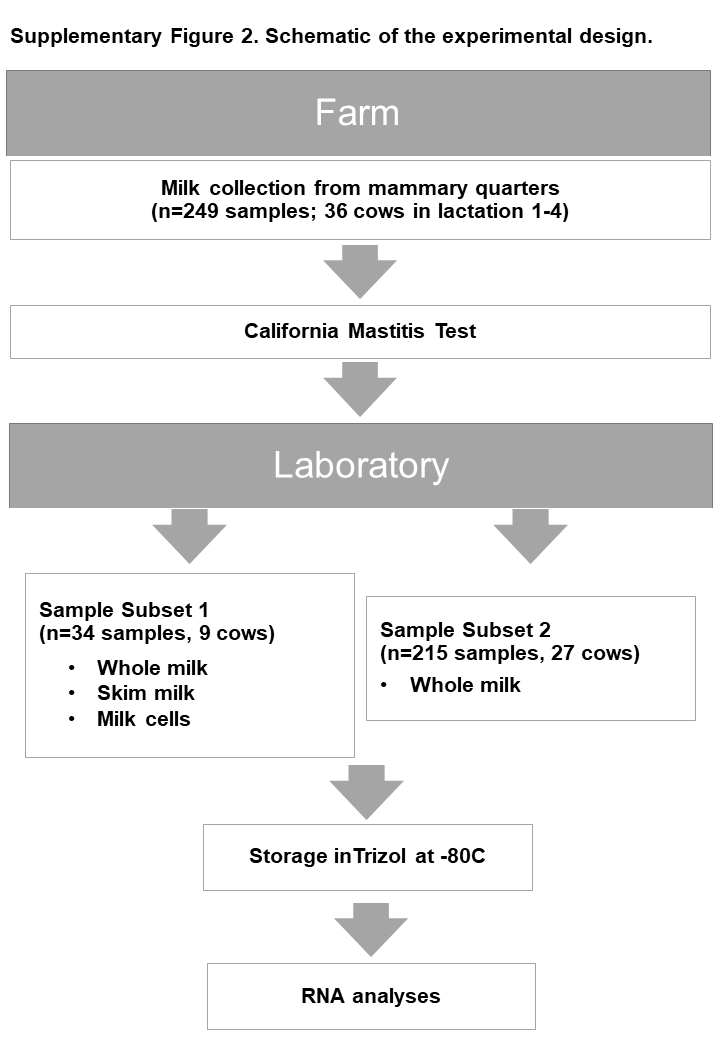

Supplement: Supplementary file 3 — Supplementary Information 3. [file 41598_2022_9214_MOESM3_ESM.tif]
